# Perceptions of Carriership of Extended-Spectrum Beta-Lactamase (ESBL)-Producing Bacteria: A Qualitative Study

**DOI:** 10.3390/nursrep14030135

**Published:** 2024-07-23

**Authors:** Karin Uggla, Robin Razmi, Josef D. Järhult, Maria Lindberg

**Affiliations:** 1 Centre for Research and Development, Uppsala University, Region Gävleborg, 801 87 Gävle, Sweden; karin.uggla@regiongavleborg.se (K.U.); robin.razmi@regiongavleborg.se (R.R.); 2 Department of Medical Sciences, Uppsala University, 751 85 Uppsala, Sweden; josef.jarhult@medsci.uu.se; 3 Department of Caring Sciences, Faculty of Health and Occupational Studies, University of Gävle, 801 76 Gävle, Sweden

**Keywords:** antimicrobial resistance, colonization, ESBL, person-centered care, qualitative research

## Abstract

The spread of antimicrobial resistance is a global health concern, and resistance mediated by Extended-Spectrum Beta-Lactamases (ESBLs) can cause major consequences. The aim of this study was to explore individuals’ perceptions of their daily life and how they cope after being diagnosed with carriage of ESBL-producing bacteria. A qualitative study was conducted with a descriptive design. Data were collected through individual interviews with 24 persons having ESBL carriership, via a semi-structured interview guide. The data were analyzed using qualitative content analysis. The informants’ perceptions on “Living with uncertainty about carriership that impacts oneself and others” were interpreted. Experiences of altered behaviors and sentiments due to ESBL carriership were described, as ESBL carriership was perceived to have a psychosocial impact on many informants. Ambiguous and inconsistent information tended to exacerbate these perceptions. The results of this study emphasize the importance of conveying individualized information, both at the time of diagnosis of ESBL carriage and thereafter. This study was not registered.

## 1. Introduction

The spread of antimicrobial resistance (AMR) is one of the greatest public health challenges today [1]. One major aspect of AMR is the emergence of beta-lactam resistance in Gram-negative bacteria of the enterobacterales order. Beta-lactam resistance is often conferred by mobile genetic elements (plasmids), which allow bacteria to express enzymes that hydrolyze the antibiotic molecule before it reaches its target. These enzymes are collectively known as beta-lactamases, among which the more potent are known as Extended-Spectrum Beta-Lactamases (ESBLs). Resistance mediated by ESBLs may have serious consequences, as it renders some of the most important antibiotics partially or completely ineffective [2]. 

The mobility of plasmids allows ESBLs to spread horizontally between bacteria, and plasmids also possess the ability to spread between different bacterial species. Accordingly, ESBL-producing bacteria have displayed the potential to cause large outbreaks, especially in hospital wards [3]. Therefore, colonization with these resistant bacteria is a notifiable condition in Sweden. Furthermore, individuals with ESBL carriage receive information about their condition, and a medical alert is created in their patient record. The duration of colonization is not clearly defined, and, in Sweden, there is currently no established practice for when an individual can be considered free from such carriage [4]. 

ESBL carriage does not impart any imperative rules of conduct upon the individual; however, non-binding medical advice is provided. Individuals are advised to inform healthcare personnel about the carriership, to observe careful hand hygiene and to avoid bathing in public pools in certain circumstances [4]. Hence, there is a potential for these individuals to be constantly reminded of their ESBL carriership. Previous studies describe a perceived lack of information about ESBLs and that individuals with carriership search for information and develop strategies to manage their diagnosis [5,6]. Concordantly, patients may experience stigmatization and negative special treatment, both from healthcare personnel and from society at large [6,7]. 

Access to adequate information enhances patients’ awareness of their own capabilities, and informed individuals report higher levels of confidence [8]. Person-centered care focuses on the patient’s perspective of illness and aims to comprehend their psychosocial context [9,10,11], allowing individuals to grasp the purpose of their treatment. This approach fosters a sense of security while reducing vulnerability [8]. A collaborative partnership between the patient and the healthcare provider is founded on a good relationship, common goals, information exchange and an understanding of the patient’s conditions and needs [9,10,11]. Active listening and identification of the patient’s concerns contribute to accurate diagnoses, facilitated trust and reduced anxiety. Conversely, insufficient communication can cause harm [12]. Thus, having a person-centered approach in patient–professional interactions is of importance for patient safety. Further knowledge about how ESBL carriage is experienced in daily life after diagnosis and how information is perceived is important for the prevention of stigmatization and negative special treatment. As the prevalence of ESBL-producing bacteria is increasing, there is a need to enlighten healthcare personnel about experiences of ESBL carriage as a foundation for guiding progress in patient care. This study aims to explore individuals’ perceptions of their daily lives and how they cope with it after their diagnosis of ESBL-producing bacteria carriage.

## 2. Materials and Methods

A descriptive design and a qualitative approach were used. 

### 2.1. Setting and Sample

This study was conducted in Gävleborg county, Sweden, a county with approximately 287,000 inhabitants. Individuals diagnosed with ESBL carriership during the period 2015–2019 and aged 18 years or older were eligible for inclusion. By using purposive sampling, potential participants were identified via a register of notifiable diseases maintained by the County Medical Officer. A research assistant telephoned 37 individuals, gave information about the study and asked for their participation. The 32 individuals who expressed an interest in participating were informed that they would be contacted by the first author via telephone so that they could receive further information, ask questions and schedule the interview. In total, 24 individuals diagnosed with ESBL carriage between 2016 and 2019 agreed to participate; see Table 1 for informant characteristics.

### 2.2. Data Collection

The first author conducted open-ended semi-structured individual interviews between September 2022 and January 2023 at a location chosen by the informant; 7 interviews were carried out at a healthcare reception, and 17 via a digital video meeting or telephone. All interviews were audio-recorded as digital audio files and stored on a password-protected server. An interview guide was used to ensure that all informants were asked all questions concerning their description of a typical day, whether the carriership had affected their life in any way and whether they had perceived any obstacles in their daily life or in contact with healthcare. When needed, clarifying questions, such as “Please tell me more about that”, were asked to obtain more in-depth information. The interviews lasted between 12 and 45 min and were 23 min long on average. Each interview was transcribed verbatim into a text file. The interviewer had no prior relationship with the informants.

### 2.3. Data Analysis

Data were analyzed using inductive manifest and latent content analysis inspired by Graneheim and Lundman’s [13] description. The first and last author read the transcripts to become familiar with the text and identified meaning units based on the study aim. The meaning units were then condensed and labelled with a code in accordance with their content. The analysis process continued by grouping the codes into subcategories based on their similarities. The manifest analysis was concluded by abstracting the subcategories into categories. To interpret any underlying meaning [14] in the transcribed interviews, the material was re-read, and a theme was formulated describing how the informants talked about the studied phenomena. Excerpts were identified to illustrate the findings. Throughout the dynamic analysis process, the first and the last authors engaged in a continuous dialogue. All authors discussed the analysis until consensus was reached.

### 2.4. Ethical Considerations

The Swedish Ethical Review Authority approved the study protocol (reg. no. 2022-03364-01; supplementary application 2022-05296-02). All informants were informed that participation was voluntary and that the care and/or treatment they were receiving would not be affected by their participation. The informants’ integrity and autonomy were ensured by presenting the results on group level. Written informed consent was obtained from all informants. The informants had the option to withdraw at any time without giving a reason and could decline to answer any question during the interview. An invitation was extended to contact the researcher with questions after the interview.

## 3. Results

The theme “Living with uncertainty about carriership that impacts oneself and others” describes the informants’ perceptions of living with ESBLs and is based on two manifest categories (see Table 2 for an overview). The informants described various perceptions of their ESBL carriage’s influence on daily life, including effects on behavior, sentiments and emotions. Moreover, they described a reluctance to talk about the carriership with others. A perceived lack of information and uncertainty about the carriage were common among the informants, indicating inadequate information provision. 

### 3.1. Changed Behaviors and Sentiments

This category includes four sub-categories that describe how the informants are reminded of their carriership, its impact on social life and how they deal with the burden of the carriage, as well as how they tell others about it. 

#### 3.1.1. Healthcare Contacts Constitute a Reminder of Carriership

Even though most of the informants recognized that they received good care and treatment from the healthcare personnel, reminders of ESBL carriership were common during healthcare interactions. During hospital care, some informants perceived an advantage in being offered a private room and toilet, while others acknowledged a feeling of isolation. If no isolation measures were conducted by the healthcare personnel during hospital care, the informants felt confused, as they had been informed of their importance. A general understanding of the need for protective measures was expressed; however, some perceived it as uncomfortable. The informants described varying degrees of hygiene measures, e.g., an initial use of protective clothing, which was later discontinued during the same episode. They also observed that their dishes were handled in a certain way, and that their appointments were scheduled last. Some informants were concerned that their carriership created an inconvenience for the healthcare personnel and expressed feelings of guilt. The informants displayed extra hygiene caution during healthcare contacts to prevent transmission and emphasized the need for consistent routines and awareness among the healthcare personnel.

*“Those who have come in look like they are from another planet, they ask ‘What’s wrong now then?’ Yes, then they have said those resistant things…. Then they changed their mind (laughter), well, I don’t know, maybe they have bad information about how to handle things”*

(Informant 7).

#### 3.1.2. The Carriership Changed Social Life

The informants felt unfortunate to have acquired ESBLs. They described refraining from social contact and activities, spending more time at home, and felt inhibited.

*“So, I have stayed away from…uh… parties and things like that. It has maybe only been after three or four years that I have tried to do anything and then, no, but I stopped drinking too. I quit all alcohol, coffee, spicy food, drinking…yes…all the fun things in life”*

(Informant 9).

Some informants described that they had stopped shaking hands due to the risk of spreading the infection. Informants that used to go swimming in pools described being prevented from it and perceived it as a reminder of their carriage. In contrast, some informants described no difference in their daily lives and did not feel hindered from any activities, which made them wonder whether they were negligent. Most informants did not experience any differences at work, and none of them felt excluded in the workplace. 

#### 3.1.3. Dealing with the Burden of Carriership and Its Emotional Influence

The constant awareness of the bacterium’s presence was a shared sentiment among the informants, with some initially shocked and saddened by the diagnosis. The informants described thinking about their ESBL carriage and how to cope with it, particularly those with recurrent urinary tract and fungal infections. As a means to prevent infection, some reported increased fluid intake. Feelings of fear and worry were commonly expressed, with concerns of infecting loved ones (especially children), smelling like urine and contracting illnesses that would require antibiotics. The informants feared that antibiotics would not help them in the future.

*“It’s in the back of my mind that the antibiotics might not work, that’s what I’m afraid of…//…it’s like life…one of the lifelines so to speak when you get sick”*

(Informant 3).

Mental health struggles, such as a diminished sense of happiness and even feelings of impending death, were described. Some experienced anxiety attacks, impacting their ability to work. The act of seeking information was also described as a source of anxiety. Some, however, described having transitioned from initial fear to acceptance, experiencing a sense of calm.

Hygiene considerations were pervasive among the informants, as was the adoption of thorough practices, such as the frequent use of a hand sanitizer, the use of personal towels and cleaning the toilet. Moreover, they described meticulous planning to avoid using public bathrooms. While some informants remained consistently cautious, others had changed their habits over time. Visiting others was perceived as stressful due to limited access to a hand sanitizer, and they always brought personal bedding. In this regard, the COVID-19 pandemic was perceived as having a normalizing effect. Owing to their concern of spreading the infection at work, some used additional protection including long gloves. The informants working in healthcare described that they felt unprofessional.

*“You are reminded all the time… that is so hard but… it’s mostly at home or if you’re going somewhere… if you go to someone’s place… then you’re also, it causes stress somehow, they don’t have hand sanitizer to the extent that you have at home… so things become very difficult”*

(Informant 16).

#### 3.1.4. Making the Decision about Disclosure

The informants described different reactions when informing others about their carriership. Many experienced no reaction at all, while others described questions, surprise, curiosity and a desire to know more. Family members sometimes reacted with concern about the risk that they themselves would be infected and that ESBLs would affect other diseases such as diabetes by altering the blood glucose level. There were differences in whom the informants chose to disclose their ESBL carriage to. Some did not tell anyone, some disclosed it to their closest friends, while others did not share this information with anyone outside the family. Owing to a fear that others would view them as careless due to their carriage, some were reluctant to disclose it to their children. The informants understood the importance of disclosing their carriage during healthcare visits and did so. However, some mentioned that they had stopped doing it, since no one listened, and some described they did not want to share the information in the waiting room. Most chose not to disclose it at work, while some who were previously unaware of the carriership said that they might share it in the workplace.

*“I’ve been very selective about who I have told, so only the immediate family… that is, the nuclear family…//… and then some friends whom I trust 100 percent”*

(Informant 14).

### 3.2. Inadequate Information Provision

This category includes two subcategories that describe the lack of information and uncertainty about lifelong carriership.

#### 3.2.1. Lack of Information

Most informants reported that the information on ESBL carriage they received from healthcare providers was inadequate and that they perceived that the healthcare personnel did not have sufficient knowledge about ESBLs. To obtain more information, some booked a telephone appointment with a doctor, but this did not provide them with any additional information.

*”…then I wanted to have a conversation with a doctor to get more information myself… We got to talk, but I didn’t know any more after we had finished talking”*

(Informant 23).

The informants received various degrees of information, ranging from basic information about hand hygiene to specific instructions on disclosing their carriage, avoiding swimming pools and securing a private hospital room. The interpretation of the information also varied, with some feeling that they could continue to live as usual, and others feeling that the carriership changed their way of life forever. The information was received via letters, phone calls or during hospital visits, sometimes on several occasions. Some informants perceived it difficult to remember the provided information due to illness at the time or owing to inattention. They requested more information and a personal contact they could turn to for unanswered questions, as well as a clearer explanation of why they should act in a specific way. 

Several informants stated that they never received information about ESBL carriage and were unaware of it before participating in the interview. They felt shocked and surprised that they did not know, finding it uncomfortable. Some informants perceived this as a failure on the part of healthcare, while some expressed curiosity.

#### 3.2.2. Ambiguity of Lifelong Carriership

Most informants perceived a lack of knowledge about their ESBL carriage and expressed a desire to know where they contracted it. To gain more knowledge, they resorted to self-education, including search engines like Google. They described it as challenging to find answers and expressed uncertainty about the reliability of the information they came across. The informants perceived ambiguity regarding the duration of their ESBL carriership and were unsure if they still had it, as the healthcare personnel had given information suggesting that they no longer did. Some asked about tests to see if ESBL carriage was still present but were informed that such testing was not possible. Many believed that extensive antibiotic use was necessary to acquire ESBL carriership, and therefore felt uncertainty about the carriage as they had not received antibiotics to such a large extent. Furthermore, the informants experienced ambiguity about the severity of the condition, especially since the healthcare personnel tended to act differently in care situations.

*“I actually got an information sheet where it talked a little bit about it, but then you Googled it yourself, you did it in the beginning before you really knew what it was… Actually, I’m really not fully aware now either … from the health care providers, there was really only one … yes, a folder, with… I think it’s something standardized that you actually send out”*

(Informant 22).

## 4. Discussion

ESBL carriership has great impact on both social life and psychological well-being for some individuals, while others are not affected at all. It is common to be reminded of and affected by the carriership during contacts with healthcare professionals. Previous studies [5,6,15] illustrated a perceived lack of information, which is reinforced by the results of this study. Many informants refrained from social contact for fear of infecting others. Even though no such advice was given, they perceived it to be easier to stay at home. Some informants were anxious about smelling like urine, which they linked to ESBLs. These perceptions could be explained by the lack of information and the uncertainty derived from self-education. In this study, the informants described a desire for more information and a personal contact to whom they could turn with unanswered questions. This is consistent with the results of Hamilton et al. [6], which showed a lack of support. Insufficient communication can cause psychological harm, including loss of trust, stress, anxiety and feelings of vulnerability [12]. Based on the informants’ perceived lack of information, it is important for the healthcare personnel to consider the patient’s well-being, to follow up on the information provided and to provide an opportunity to ask questions. A lack of information may lead to a sense of stigmatization [5]. Hamilton et al. [6] reported in their study that individuals with AMR experience both fear and stigma. In a review by Rump et al. [16] focusing on MRSA carriage, perceived stigma was present in more than half of the cases and was most pronounced in relation to healthcare encounters. However, Wijnakker et al. [17] concluded that most participants exposed to isolation during hospital care did not experience stigma owing to AMR bacteria. Since there is no follow-up of ESBL carriage, such feelings will not be revealed to the healthcare personnel. Given the perceived lack of knowledge among the healthcare personnel and the patients themselves, it is valuable that healthcare encounters are based on a person-centered approach which could reduce the perceived stigma. Havana et al. [9] found that person-centered care promotes a sense of security and reduces vulnerability. A person-centered approach emphasizes individualized information based on a trustful relationship and an understanding of the psychosocial context [9,10,11]. 

Many informants in the present study stated that they were not aware of their ESBL carriage. This corresponds with the results of Wiklund et al. [18], who described inadequate information provision and that some informants did not receive any information at all. Lindberg et al. [19] also demonstrated a lack of information among patients carrying AMR bacteria, suggesting that this phenomenon is not new. In the present study, the informants may not have received information or may have received it without understanding it, and the healthcare staff may not have followed up to determine the informants’ understanding. There were also significant differences in terms of the informants’ degree of concern. This ranged from no concern at all to anxiety and thoughts of death, which is consistent with previous research [5,6]. Several informants found the duration of the carriage to be unclear, and some were informed that they were no longer ESBL-producing bacteria-colonized, despite the absence of a national consensus as to when an individual can be considered free from ESBL-producing bacteria. The different experiences are important to bear in mind in healthcare encounters. This is supported by Hamilton et al. [6], who found that individuals with AMR carriage lacked support from the healthcare personnel. The informants in the present study stated that the healthcare personnel would sometimes take excessive protective measures. This may be justified to some degree by the setting and the presence of risk factors, as well as by the protective behavior adopted by society as a whole during the COVID-19 pandemic. These factors, however, are unlikely to fully explain the differences described by the informants. A lack of knowledge among the healthcare personnel may also be a contributing factor, since, according to Wiklund et al. [18], the healthcare personnel report having poor knowledge about ESBLs. Aldrazei et al. [20] described uncertainty among the healthcare personnel about procedures and policies, emphasizing the importance of continuing education. The informants described a fear that antibiotics would not help if they were to become ill. These thoughts could arise due to uncertainty, which creates a sense of dependence. This further emphasizes the need for individualized information to minimize the impact on individuals with ESBL carriership. Working in accordance with person-centered care [9,10] is thus vital. 

### Methodological Considerations

Credibility, dependability and transferability [13,14] were considered in this qualitative interview study. To enhance credibility, the methodological sections of setting and sample, data collection and data analysis were well defined in concordance with the research objective. Excerpts from the interviews were used to reinforce the results. To further enhance credibility and dependability, the researchers maintained a constant dialogue to achieve agreement in the analysis process. Dependability was also maintained by conducting interviews within a limited time frame and by using an interview guide to ensure that all questions were asked to all informants. The interviews could be considered short, yet the studied phenomena were well defined, and the responses were assessed as informative. With this thorough methodological description, transferability was addressed; however, the final judgment is at the discretion of the reader.

## 5. Conclusions

ESBL carriership is associated with significant side effects, inducing negative thoughts and feelings among those afflicted. These effects can be alleviated by providing thorough and individualized information, both at the time of diagnosis and afterwards. Such is rarely the case, however, as many informants described inadequate communication from the healthcare personnel. ESBL prevalence is expected to rise; it will therefore become increasingly important to provide the information that individuals with carriership need in order for their lives to be impacted minimally.

## Figures and Tables

**Table 1:** Characteristics of the participating informants.

Informant Characteristics	N = 24
Gender (number)	
Women	14
Men	10
Awareness (number)	
Knowledge of carriage at the time of interview	17
No knowledge of carriage at the time of interview	7
Civil status (number)	
Partner or married	18
Single	6
Work situation (number)	
Retired or disability retirement	7
Student	1
Employed	15
Unemployed	1
Education (number)	
Elementary school	2
Upper secondary school	15
University	7
	Min–Max
Age (years)	19–72

**Table 2:** Overview of subcategories, categories and theme following the content analysis.

Theme	Category	Subcategory
Living with uncertainty about carriership that impacts oneself and others	Changed behaviors and sentiments	Healthcare contacts constitute a reminder of carriership
		The carriership changed social life
		Dealing with the burden of carriership and its emotional influence
		Making the decision about disclosure
	Inadequate information provision	Lack of information
		Ambiguity of lifelong carriership

## Data Availability

The participants in this study did not give written consent for their data to be shared publicly; so, due to legal restrictions, the data are not publicly available.

## Public Involvement Statement

There was no public involvement in any aspect of this research.

## Guidelines and Standards Statement

This manuscript was drafted following the Standards for Reporting Qualitative Research (SRQR) for qualitative research.

## References

[1] World Health Organization (2015). Global Action Plan on Antimicrobial Resistance [Internet].

[2] Peleg A.Y., Hooper D.C. (2010). Hospital-Acquired Infections Due to Gram-Negative Bacteria. N. Engl. J. Med..

[3] Bush K., Fisher J.F. (2011). Epidemiological Expansion, Structural Studies, and Clinical Challenges of New β-Lactamases from Gram-Negative Bacteria. Annu. Rev. Microbiol..

[4] Smittskyddsläkarföreningen (2023). Information for Doctors Regarding ESBL (Swedish). Smittskyddsläkarföreningen. https://slf.se/smittskyddslakarforeningen/app/uploads/2023/04/esbl-lakarinformation-2023-04-18.pdf.

[5] Wiklund S., Örtqvist Å., Berlin A., Stamm C., Broliden K. (2018). Experiences and consequences of living with extended-spectrum betalactamase-producing bacteria: A qualitative study. Am. J. Infect. Control.

[6] Hamilton R.A., Lond B., Wilde L., Williamson I. (2024). Understanding the lived-experience and support-needs of people living with antimicrobial resistance in the UK through interpretative phenomenological analysis. Sci. Rep..

[7] Wiklund S., Hallberg U., Kahlmeter G., Tammelin A. (2013). Living with extended-spectrum β-lactamase: A qualitative study of patient experiences. Am. J. Infect. Control.

[8] Havana T., Kuha S., Laukka E., Kanste O. (2023). Patients’ experiences of patient-centred care in hospital setting: A systematic review of qualitative studies. Scand. J. Caring Sci..

[9] Ekman I., Swedberg K., Taft C., Lindseth A., Norberg A., Brink E., Carlsson J., Dahlin-Ivanoff S., Johansson I.-L., Kjellgren K. (2011). Person-centered care—Ready for prime time. Eur. J. Cardiovasc. Nurs..

[10] Britten N., Moore L., Lydahl D., Naldemirci O., Elam M., Wolf A. (2016). Elaboration of the Gothenburg model of person-centred care. Health Expect..

[11] Constand M.K., MacDermid J., Bello-Haas V.D., Law M. (2014). Scoping review of patient-centered care approaches in healthcare. BMC Health Serv. Res..

[12] Hernan A.L., Giles S.J., Fuller J., Johnson J.K., Walker C., Dunbar J.A. (2015). Patient and carer identified factors which contribute to safety incident in primary care: A qualitative study. BMJ Qual. Saf..

[13] Graneheim U.H., Lundman B. (2004). Qualitative content analysis in nursing research: Concepts, procedures and measures to achieve trustworthiness. Nurse Educ. Today.

[14] Graneheim U.H., Lindgren B.-M., Lundman B. (2017). Methodological challenges in qualitative content analysis: A discussion paper. Nurse Educ. Today.

[15] Gaube S., Däumling S., Biebl I., Rath A., Caplunik-Pratsch A., Schneider-Brachert W. (2023). Patient with multi-drug-resistant organisms feel inadequately informed about their status: Adverse effects of contact isolation. J. Hosp. Infect..

[16] Rump B., Boer De M., Reis R., Wassenberg M., Van Steenbergen J. (2017). Signs of stigma and poor mental health among carriers of MRSA. J. Hosp. Infect..

[17] Wijnakker R., Lambregts M.M.C., Rump B., Veldkamp K.E., Reis R., Visser L.G., de Boer M.G.J. (2020). Limited multi-drug resistant organism related stigma in carriers exposed to isolation precautions: An exploratory quantitative questionnaire study. J. Hosp. Infect..

[18] Wiklund S., Fagerberg I., Örtqvist Å., Broliden K., Tammelin A. (2015). Staff experiences of caring for patients with extended-spectrum β-lactamase-producing bacteria: A qualitative study. Am. J. Infect. Control.

[19] Lindberg M., Carlsson M., Högman M., Skytt B. (2009). Suffering from meticillin-resistant Staphylococcus arueus: Experiences and understandings of colonisation. J. Hosp. Infect..

[20] Aldrazei F.A., Rabaan A.A., Alsuliman S.A., Aldrazi H.A., Alabdalslam M.H., Alsadiq S.A., Alhani H.M., Bueid A.S. (2020). ESBL expression and antibiotic resistance patterns in a hospital in Saudi Arabia: Do healthcare staff have the whole picture?. J. Infect. Public Health.

